# Ras-related protein Rap2c promotes the migration and invasion of human osteosarcoma cells

**DOI:** 10.3892/ol.2021.12723

**Published:** 2021-04-12

**Authors:** Jinxia Wu, Wenqi Du, Xiucun Wang, Lulu Wei, Yaojie Pan, Xiaojin Wu, Jinling Zhang, Dongsheng Pei

Oncol Lett 15: 5352-5358, 2018; DOI: 10.3892/ol.2018.7987

Subsequently to the publication of this paper, an interested reader drew to the authors’ attention that two pairs of data panels in Fig. 3C appeared to contain overlapping data (comparing between the si-Ctrl/si-Rap2c#2 and the Vector/Rap2c panels).

The authors have re-examined their original data and realized that the Vector and the Rap2c data panels for Fig. 3C were selected incorrectly. The revised version of Fig. 3, containing the correct data for the Vector and the Rap2c experiments in Fig. 3C, is shown below. The authors regret the inadvertent errors that were made in the preparation of the published figure, and confirm that these errors did not seriously affect the conclusions reported in the paper. The authors are grateful to the editor of *Oncology Letters* for allowing them the opportunity to publish a Corrigendum, and all the authors agree to this Corrigendum. Furthermore, they apologize to the readership for any inconvenience caused.

## Figures and Tables

**Figure 3.**
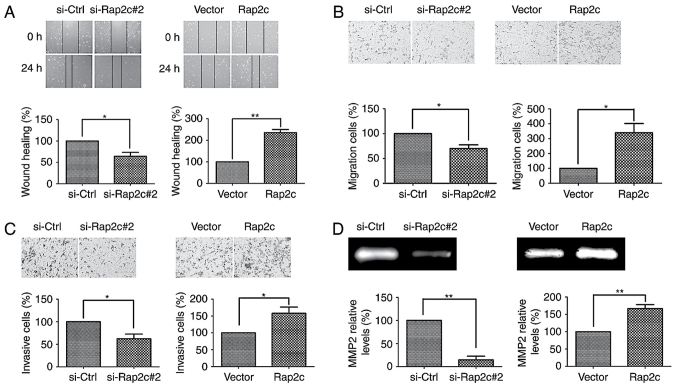
Effect of Rap2c overexpression and knockdown on the invasion and migration of U2OS cells. (A) Wound-healing assays were performed in U2OS cells transfected with a Rap2c expression plasmid or siRNA (magnification, ×100). *P<0.05 vs. si-Ctrl; **P<0.01 vs. Vector. (B) Cell migration was performed following transfection with siRNA or a pcDNA3.1-Rap2c expression plasmid (magnification, ×100). *P<0.05 vs. si-Ctrl or Vector. (C) Cell invasion was measured following transfection with Rap2c expression plasmids or siRNA (magnification, ×100). *P<0.05 vs. si-Ctrl or Vector. (D) The relative enzyme activity of cleaved-MMP2 was measured by a gelatin zymography assay following transfection with siRNA or a Rap2c expression plasmid. **P<0.01 vs. si-Ctrl or Vector. siRNA, small interfering RNA; MMP2, matrix metalloproteinase-2; Ctrl, control; Vector, empty vector.

